# Exploring the impact of functional, symbolic, and experiential image on approach behaviors among state-park tourists from India, Korea, and the USA

**DOI:** 10.1057/s41599-023-01527-y

**Published:** 2023-01-27

**Authors:** Nripendra Singh, Jongsik Yu, Antonio Ariza-Montes, Heesup Han

**Affiliations:** 1 Pennsylvania Western University, Clarion, PA USA; 2 Cheongju University, Cheongju, Korea; 3 Universidad Loyola Andalucía, Córdoba, Spain; 4 Sejong University, Seoul, Korea

**Keywords:** Psychology, Business and management

## Abstract

This study provides insights into the influence of state park image, visitor emotions, and place identity on visitors’ revisit intentions by considering the moderating impact of national culture. A quantitative process with the data collected in India, Korea, and the US was used. Hierarchical regression analysis evidences the moderating role of national culture, which is hardly explored in the state park context. Results confirm that most hypotheses are fully or partially accepted, which suggests that brand image and national culture influence visitor intention. This study helps practitioners better understand the relevance of national culture in developing appropriate visitor attraction/retention strategies.

## Introduction

Marketing of national and state parks is an important, rapidly emerging area of research within the tourism discipline due to its importance to the economy and communities (Perry et al., 2021). State parks provide for special-interest tourism (Nogueira and Pinho, 2015) and act as the key component to the destination’s image and competitiveness. State parks attract domestic and international visitors as they provide unique experiences and a great deal of value-in-use activities such as picnicking, hiking, wildlife watching, and fishing. Additionally, they are great places for outdoor recreation, entertainment, and escapism. From the brand perspective, state parks can be grouped together to form an umbrella brand for multiple benefits to all stakeholders (Dosen et al., 1994). The term state park will be used to represent both national and state parks in this paper.

There are more than 4000 national/state parks in the world (Harford, 2021) attracting millions of visitors every year, contributing to the economy through jobs, revenue creation, (Fredman and Yuan, 2011), and providing communities with nature-based entertainment and outdoor recreation (Whiting et al., 2017). For example, the combined impact of the US state parks on the economy with more than 400 state parks, covering 84 million acres of land, 17,000 miles of trails, and 221,000 campsites, and employing more than 22,000 staff (NPS, n.d.; Singh and Kealey, 2019) and 221,000 volunteers to provide services to over 280 million visitors annually (Blanck et al., 2012) is billions of dollars. State park visitors’ spending resulted in a $41.7 billion benefit to the nation’s economy and supported 340,500 jobs (DOI, 2020).

Despite the valuable contributions made to the economy and community through job and revenue creation, state parks have not been recognized as marketable resources (Eagles, 2014) until the recent past. Some researchers attributed this to the general attitude that resource preservation was a priority (Hogenauer, 2002) and the lack of emphasis on the marketing of public sector organizations (Archer and Wearing, 2002). Though things are now changing due to recent concerns for sustainability and high tourism demand. Researchers suggest a strategic approach to market state parks for sustainable tourism to benefit all stakeholders including visitors and the natural environment (López-Sanz et al., 2021b; Mayer et al., 2010). It is for these reasons that research on the marketing of state parks has gained traction in recent years (Douglas, 2016; Eagles, 2014; Mercer, 2022). Understanding the role of brand image and its connection with visitors’ emotions and place attachment has not been deeply studied. For example, while advertisements help in creating a brand image to influence behavior, it is important for managers to understand which category of the image is more appropriate for state parks to influence visitors’ revisit intentions.

Therefore, this study has three main objectives, which are as follows. The brand image helps communicate product’s core values to its customers by engaging their emotions and evoking personal beliefs (Fredman and Yuan, 2011). Advertising is a tool that creates a brand image that develops attitude because advertising commonly transfers viewer perception of the products and influences behavior (Han et al., 2019). If the state parks meet the expectations created by the ads, the visitors’ will have positive emotions and will identify themselves with it, which increases their likelihood of re-visiting it. Therefore, the first objective of this study is to understand the relationship between different categories of brand images (functional, symbolic, and experiential) on visitor emotions (pleasure and arousal).

From the literature on destination branding, it is evident that visitors emotionally attach themselves to places. Place attachment is a key factor related to visitors’ revisit intention. It has two prominent components, place identity and place dependence (Ispas et al., 2021). Groshong et al. (2020) define place identity as the emotional or symbolic importance of a place, while place dependence is related to functionality and usability. As this study does not focus on the functionality aspect of state parks, only place identity is utilized to assess the place attachment. Visitors identify themselves with the characteristics of the place and revisit places with which they get inspired/encouraged. Therefore, the second objective of this study is to understand the relationship between visitor emotions (pleasure and arousal) on place identity.

International visitors contribute substantially to the economy (Manzoor et al., 2019) Visitors differ in how they get influenced depending on cultural and other aspects such as demographic and psychographic profiles (You et al., 2000). State parks attract visitors from different parts of the world, and they may differ in their choices and influences depending on their cultural backgrounds (Vespestad and Mehmetoglu, 2010). Understanding the role of cultural differences (or national culture) on key influencers of revisit intention towards state parks can be an important contribution of this study. This focus is essential because cultures are not stagnant (Herrmann and Heitmann, 2006). Studies on international travelers have covered their motivations such as escapism to simply learn the local ecosystem (Manzoor et al., 2019; Wen and Ximing, 2008) and to seek out novelty and experiences (e.g., Manrai and Manrai, 2011). None of the studies on international travelers has examined how national culture influences the relationship between brand image, emotions, place identity, and revisiting intention to state parks. Therefore, the third objective of this study is to understand the moderating effect of the national culture on the relationships between brand image, emotions, place identity, and revisit intention to state parks.

## Literature review

### Brand image (functional, symbolic, and experiential)

Brand image is an important area of marketing research (Topcuoglu et al., 2022) and conveying a brand image to a target market is considered fundamental marketing activity (Tavitiyaman et al., 2021) since brand image indicates “how consumers feel about a brand and whether a positive relationship exists between the brand and consumers” (Plumeyer et al., 2019, p. 228). Dobni and Zinkhan (1990) defined brand image as a meaning that consumers associate with the product based on their experiences, impressions, and perceptions about the three benefits of a particular brand: functional, emotional, and symbolic. Most research studies on brand image in a variety of contexts found agreement with the above definition and concluded that functional, emotional, and symbolic images created in the minds of consumers are the reason individuals attach themselves to brands (Zaman and Aktan, 2021; Zha et al., 2020). Researchers used many different terms for ‘brand image’ such as brand associations, brand performance, brand imagery, green brand image, and brand love (Andreini et al., 2018; Chen, 2010; Eklund, 2022; Junaid et al., 2019). Advertising and promotional strategies can enhance brand/product knowledge, awareness, brand associations of brand image, draw emotions, create attitudes/experiences, and customer loyalty (or purchase decisions) (Hsieh and Li, 2008). Thus, it makes economic sense for brands to invest in branding strategies. Similarly, destination branding is used for creating a positive image in the minds of tourists to attach them to places. Literature from the tourism discipline reveals that branding techniques are utilized to create destination brands to attract tourists via destination images. Destination images are the sum of beliefs, emotions, and impressions tourists develop about a place (Vera and Chang, 2022). The most common and highly accepted attributes of the destination image are found to be cognitive, affective, and conative (Michael et al., 2018; Moon and Han, 2019). As the name suggests, cognitive refers to how tourists perceive a place and affective refers to how tourists feel about it, while conative refers to the behavioral aspect of tourists or their intention to visit or revisit a place. There are numerous studies on destination image that validate the influence of cognitive and affective attributes on conative attributes. However, as Kim and Yoon (2003) pointed out affective attribute has not been thoroughly studied as a cognitive attribute, which according to many studies has a major role in forming a destination image in the minds of tourists (Lehto et al., 2014). Moreover, the place in this study is ‘state parks’, which has not been studied in this context before. As ‘state parks’ does not have huge differentiation from each other even if they are in different countries and continents, the influence of ‘proximity of place’ on destination image (Martins, 2015; Stepchenkova and Mills, 2010) will not be applicable in this context. Thus, it is important to study the relationship between images and the emotions that are formed as a result of affective attributes (Vera and Chang, 2022). For the purpose of this study, the concept of image is borrowed from ‘Brand Concept Management’ (Park et al., 1986).

As explained by Park et al. (1986) in ‘Brand Concept Management’, there are three basic components derived from consumer needs: functional needs, symbolic needs, and experiential needs. Functional needs motivate the search for products to solve consumption-related problems such as solving a current problem. Functional concept in a product solves externally generated consumption needs. Symbolic needs fulfill internally generated needs by using a product such as ego-identification. Symbolic concept in a product fulfills the self-image need of the consumer. Experiential needs on the other hand refer to sensory pleasure created by the products. Experiential concept in a product fulfills internally generated needs for stimulation and/or variety. Gómez‑Rico et al. (2022) postulated that the three concepts: functional, symbolic, and experiential, create an image in a brand and that any product can be positioned with functional, symbolic, and experiential images. Different images can be used to develop different marketing strategies to attract visitors from different cultures (Al-Ansi and Han, 2019). For example, a state park can use functional images to fulfill visitors’ consumption needs such as value-in-use activities (e.g., picnicking, hiking, wildlife watching, and fishing). Plus, functional images can portray state parks as great places for outdoor recreation, entertainment, and escapism. Symbolic images can be used to emphasize exclusivity to their visitors like being an environmentally friendly or sustainable person. Experiential images may convey messages such as rejuvenation, wellness, meditation, and even knowledge enrichment. Depending on the target segment, state parks can use multiple images to create an impression in the minds of potential visitors.

### Emotions (pleasure and arousal)

Emotion is defined as people’s state of feelings and has three basic dimensions, including pleasure, arousal, and dominance (Mehrabian and Russell, 1974), which arise from cognitive appraisals of events. Two dimensions: pleasure and arousal are considered fundamental (Russell and Steiger, 1982) and sufficient to explain the environmental effect (Russell and Pratt, 1980). In the psychology literature, emotions are represented by positive and negative dimensions (Lee and Jeong, 2021). Woosnam and Norman (2010), based on the initial workings of Durkheim ([1915] 1995), considered emotional solidarity as the degree of identification with someone else or affective bonds between individuals marked by a degree of closeness and contact. According to Mehrabian and Russell’s (1974) ‘stimulus-organism-response model’ emotion is affected by various stimuli in the environment causing a response that is either approach or avoidance behavior. Emotions have been studied in various contexts to examine the relationships between different variables such as customer experiences and behavioral intention. For example, restaurants (Han et al., 2019), hotels (Trang et al., 2019), places (Hosany et al., 2017), brand attachment (Orth et al., 2012), shopping, retailing, tourism (Han et al., 2017), festivals (Lee and Kyle, 2012). Most studies have validated the findings that emotions influence the behavioral intention of consumers/visitors either via satisfaction or loyalty (Doctor et al., 2021).

In the Mehrabian and Russell Model of Affect, the feeling of pleasure is conceived as a continuum from extreme happiness to unhappiness (Russell and Mehrabian, 1977). Pleasure was initially described in terms of positive or negative feelings using adjectives such as happy–unhappy and pleased–annoyed. Arousal was described as a combination of mental alertness and physical activity such as ‘sleep, inactivity, boredom and relaxation’ versus ‘wakefulness, bodily tension, strenuous exercise, and concentration’. Russell and Carroll (1999) defined arousal using ‘ascending order of level of feelings: active, alert, attentive, and excited’ (Han et al., 2019, p. 5). Depending on the level of intensity in arousal, pleasure can have different impacts on a customer (Bakker et al., 2014). Wirtz et al. (2000) postulated that arousal can amplify the influence of pleasure on the perceived value of a product and overall satisfaction.

Emotions are ubiquitous in tourism as it relates to experiences. Past research has established that people develop relationships with places and have emotional responses toward the immediate environment (Tasci et al., 2022). Tourist’s emotions determine behavioral intention (Joo et al., 2018). Regardless of the importance of emotions in tourism research, there are few studies that assess the relationship between emotions and place attachment (Orth et al., 2012; Patwardhan et al., 2019; Woosnam et al., 2020).

### Place attachment

Research studies suggest that consumers attach themselves to situations, objects, and entities (Hosany et al., 2017) such as brands, possessions, festivals, hot spring resorts, destinations, heritage sites, natural areas, and places (Eslami et al., 2019; López-Sanz et al., 2021b). Place attachment is a developmental theory as it still lacks enough research evidence to be considered a systematic theory (Hosany et al., 2017; Scannell and Gifford, 2014), which is also reflected in the different measurement approaches used in past: unidimensional to multidimensional approaches (Tasci et al., 2022). Researchers draw from the theories of psychology and refer to place attachments as a meaningful affective bond between individuals and places (Woosnam et al., 2020). Place attachment is determined by visitors’ response to his/her memories and associations that they experience at a place, which can be both emotional and cognitive (Jorgensen and Stedman, 2001; Patwardhan et al., 2020). Place attachment is commonly used as a two-dimensions construct: place identity and place dependence. A place’s identity is rooted in the emotions created and the symbolic importance associated with it (Groshong et al., 2020), and as such, is both cognitive and personal. Place identity is about one’s assimilation of a place into his/her larger concept of self (Proshansky et al., 1983) or self-identity (Kyle et al., 2004). In other words, a place is made important and set apart from others in one’s mind by the bond that one feel (Ispas et al., 2021; Yang et al., 2022). Thus, place identity is more about symbolic importance related to emotional and relationship aspects of a place that provide meaning and purpose to one’s life (Williams and Vaske, 2003). Place dependence, also known as “functional attachment,” refers to the ability of a place to satisfy one’s needs and goals (Stokols and Shumaker, 1981; Tsai, 2012). This dimension of attachment is based on the functional or transactional approach, which is specific to the place and its resources for one’s chosen activities (Scannell and Gifford, 2014). As the purpose of this study is to assess the symbolic aspect of attachment and not the functional aspect, place attachment was measured as one dimension in this study using only place identity. Place attachment has been studied in many contexts including state parks (Hwang et al., 2005; Ramkissoon et al., 2013; Tonge et al., 2015), but the influence of emotions on place attachment and its mediating role between emotions and revisit intention has not been paid much attention (Hosany et al., 2017; Orth et al., 2012). Additionally, there is enough empirical support for the effect of place attachment on consumers’ decision-making (e.g., Tasci et al., 2022; Yan and Halpenny, 2019). In line with Prayag and Ryan (2012) call for further research examining the relationship between place attachment and intention, this study will assess the influence of place identity on behavioral intention to revisit state parks.

In light of the above discussions, we developed the following hypotheses:

H1: Functional image positively and significantly affects pleasure

H2: Symbolic image positively and significantly affects pleasure

H3: Experimental image positively and significantly affects pleasure

H4: Functional image positively and significantly affects arousal

H5: Symbolic image positively and significantly affects arousal

H6: Experimental image positively and significantly affects arousal

H7: Pleasure positively and significantly affects place identity/attachment

H8: Arousal positively and significantly affects place identity/attachment

H9: Place identity/attachment positively and significantly affects revisit intention

### National culture

A country’s culture (or national culture) has long been seen as a key attribute for behavioral differences among consumers and critical for business success (Dang-Van et al., 2020; Nguyen and Pervan, 2020; Raza et al., 2020). Cultural values, norms, and beliefs shape individual perceptions, dispositions, and behaviors (Abdelmoety et al., 2022; Torelli et al., 2020). Failure to recognize cultural differences may lead to business failures, and their understanding and adoption in developing appropriate marketing strategies may bring success (Nakata and Sivakumar, 2001). Paying attention to national culture in promoting international tourism is especially helpful economically. Moreover, as competition in tourism is rising, cultural understanding is a must for destination managers and policymakers to attract international tourists. While there is a rise in countries promoting nature as their (destination’s) asset, there is a lack of research to provide adequate marketing inputs to destination managers, especially cross-cultural differences (Vespestad and Mehmetoglu, 2010). In a recent study by Lee et al. (2022) authors found that individualism, uncertainty avoidance, and long-term orientation have a positive influence on the green economy, while cultures dominated by power distance, masculinity, and indulgence have a negative impact on the green economy. Thus, cultural components are distinct from nation to nation (Wilkins et al., 2017) and pose huge challenges for marketers (Shankar, 2020).

National culture is a potential driver of consumer behaviors and attitudes in various consumption contexts (Craig and Douglas, 2006; Shavitt et al., 2006), including tourism (Kozak, 2002; Moscardo, 2004). National culture is defined as patterns of thinking, feeling, and behaving that are rooted in the common values of a society (Nakata and Sivakumar, 2001; Wan and Nakayama, 2022). Hofstede (1997) identified six cultural factors (or values) that are universal but are observed in varying degrees in societies. These are detailed in the following paragraphs.

*Individualism/Collectivism* is the degree of distance in social relationships. Highly individualistic societies such as most Western and North European countries have distant relationships and individuals are satisfied by achieving their goals and pursuing self-interest. While those in collectivistic societies such as Asian, South European, and Latin American countries tend to have close relationships and are recognized as members of their groups (Farahani and Mohamed, 2011; Shavitt et al., 2006). As per Hofstede (2001, 1997), Western cultures tend to be individualistic whereas Asian collectivist, which suggests why Korean and Indian tourists seek travel suggestions from travel agencies and corporate travel offices, while US tourists prefer broader sources of information such as tourist information offices and the Internet to make their traveling decisions (Chen, 2000). Past research on pro-environmental behavior (Kim and Choi, 2005), service assessment (Reichert and Gill, 2004), and sports and recreational services (De Mooij, 2004) show the influence of individualism (national culture) on behavioral change (decision-making).

*Uncertainty avoidance* refers to the level of discomfort with the unknowns in the future. Those from higher uncertainty avoidance cultures (e.g., Korea) follow rules and do not take risks in order to control uncontrollable/unknown situations. On the contrary, people from lower uncertainty avoidance cultures (e.g., India) tend to accept risk and reflect flexible behavior to manage different situations (Hofstede, 2001). Money and Crotts (2003) postulated that visitors from countries with low uncertainty avoidance tend to spend more time on information search about the destinations. Tourists from high uncertainty avoidance cultures (such as Japan and Korea) seem to engage in uncertainty-reducing behaviors. An example of this purchasing behavior includes getting information from tour companies, travel offices, guides, and from friends (Chen, 2000). They tend to purchase prepaid tour packages, stay for shorter periods, travel in larger groups, and visit fewer destinations as compared with low uncertainty-avoidance cultures (such as US, or India), who prefer visiting new and unique destinations during international travel (Reisinger and Crotts, 2009).

*Power distance* is the extent to which authority and social disparities are acceptable such as wealth and position (Torelli et al., 2020). For example, Indian society has a strongly hierarchical and bureaucratic social structure, so much so that ‘privileges or disadvantages are transmitted by inheritance’ (Moran et al., 2014, p. 372) like Japan. Such cultures have strong dependency relationships within the family, the workplace, and even in educational systems (Farahani and Mohamed, 2011). Culture in the US and similar less power-distanced countries promote not being dependent on others, which is reflected in studies on tipping and travelling behavior among tourists (Michael et al., 1993). Power distance impact service perception (Wirtz et al., 2000) e.g., tourists from the US may value intangible aspects such as ‘made to feel welcome’ important as compared to visitors from India, who might look for dependability and efficiency (Tsang and Ap, 2007).

*Masculinity* is the inclination to value success, assertion, and accomplishment. Masculine societies (e.g., US) highlight materialistic gains, while feminine focus on relationships, nurturance, and life quality (e.g., Korea). Showing success or being a winner is not given importance and is even considered negative. Crotts and Erdmann (2000) postulated that visitors from highly masculine societies tend to report dissatisfaction more than less masculine societies. In other words, visitors from countries where assertive behavior is encouraged are less sympathetic toward others and are highly judgmental.

*Long-term orientation* signifies ‘care for future’. It is reflected in the values of thrift, loyalty, and saving (e.g., Korea). Short-term orientation focuses on now or near term (e.g., US). (Reisinger and Crotts, 2009) suggested that visitors from Asian countries have a short-term view of attractions, entertainment, services, and activities, which reflects their orientation toward savings. Alcantara-Pilar and Barrio-Garcia (2015) found that this orientation is influential in changing attitudes toward tourist destination websites. Culture with low orientation forms positive attitudes due to usefulness, while people with high orientation feel positive due to satisfaction. Long-term orientation impacts consumer decision-making (Bearden et al., 2006).

*Indulgence* refers to societies that allow free gratification of basic/natural human drives related to enjoyment in life (e.g., US), while restraint societies suppress gratification of needs by strict social norms (e.g., India). Indulgence and restraint values can be very useful in tourism. For example, visitors from indulgence-oriented cultures (such as the US) tend to remember positive emotions and experiences as compared to restraint cultures (e.g., India and Korea), who tend to remember negative experiences, which impact post-purchase evaluations (Koc et al., 2017). Therefore, tourists from India and Korea may tend to be less satisfied with hospitality services as compared to visitors from the US (Su et al., 2016). Based on the above discussion, the following hypothesis is proposed:

H10a-i: National culture has a significant moderating impact on the relationships between image dimensions, pleasure, arousal, place identity, and revisit intention.

**Fig. 1:**
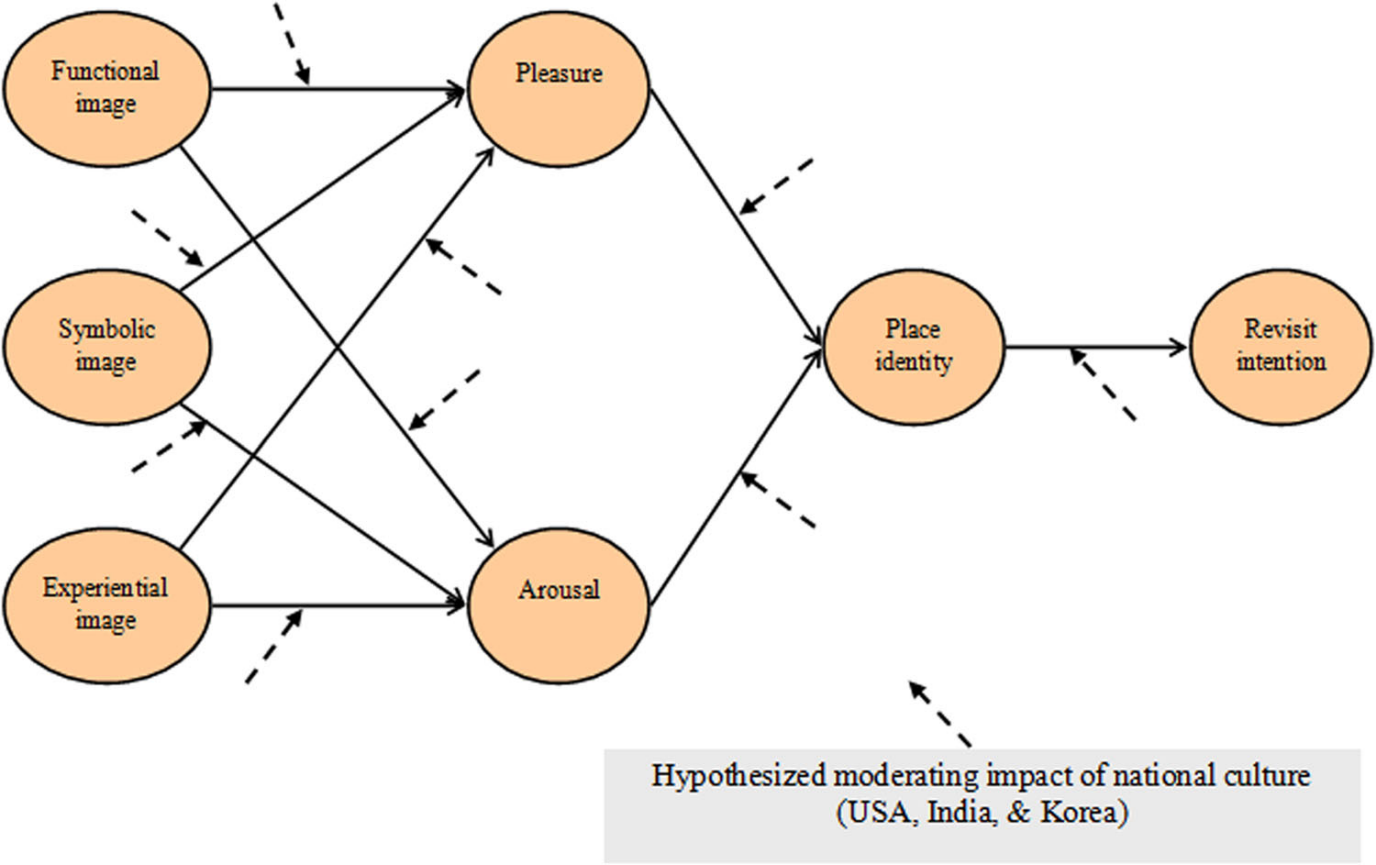
Proposed theoretical model for State-park tourists’ approach behaviors.

As discussed above, it is important to understand the cultural differences in the context of tourism and to understand its influence on the relationships between different variables in this study as shown in Fig. 1.

The decision to get data from three countries India, the US, and Korea is not only based on convenience but also because these countries together represent all six cultural dimensions in both high and low categories. India represents a culture with a high-power index, moderate individualism, masculinity, uncertainty avoidance, a long-term orientation, and low indulgence. Korea represents a culture that has very high long-term orientation and uncertainty avoidance traits, slightly high to moderate power distance and masculinity, low on indulgence, and very low on individualism. The culture of the US can be represented by very high individualism, high indulgence and masculinity, moderate power distance and uncertainty avoidance, and low long-term orientation. The above definitions are based on the scores provided by Hofstede’s national culture indices (https://www.hofstede-insights.com/) (see Table 1).

**Table 1:** Hofstede’s national culture indices.

	India	Korea	USA
Power distance	77	60	40
Individualism	48	18	91
Masculinity	56	39	62
Uncertainty avoidance	40	85	46
Long-term orientation	51	100	26
Indulgence	26	29	68

## Method

### Measures

The survey instrument for this study was developed using validated measures. The measurement items were adapted from existing scales in the area of consumer behavior, hospitality, tourism, and marketing after an extensive literature review (Arklina et al., 2020; Groshong et al., 2020; Han et al., 2019; Ispas et al., 2021; Kim and Kim, 2011; Paek et al., 2012; Park et al., 1986; Song et al., 2015; Tsaur et al., 2007; Wynveen et al., 2020). All constructs were measured with multiple items (see Supplementary Information). A total of 23 items were measured under seven dimensions within four constructs, using a 5-point Likert scale (1 = strongly disagree to 5 = strongly agree). In particular, brand image was measured using three dimensions (functional image, symbolic image, and experiential image). A total of 12 items were measured; the functional image had five items, the symbolic image had three items, and the experiential image had four items. Emotions were measured using six items under two dimensions, pleasure and arousal. Place attachment was measured using one dimension, place identity, with two items. Revisit intention was measured using three items as suggested in the literature. The questionnaire, including these measurement items, was pre-tested with marketing and hospitality academics and industry experts' review.

### Data collection

In this study, data was collected from the respondents from three countries—India, Korea, and the United States—using a purposeful method. A total of 812 usable responses were found from 1400 collected. Out of 550 responses from India 191 were found usable (35%). Similarly, 404 usable responses from 550 in the USA (74% usable) and about 217 usable responses from 290 in Korea (75% usable). The survey instrument was pilot tested and reviewed by at least one expert from each country before collecting the final data. The expert was identified based on the qualification, academic and research experience, and reputation in the discipline. Minor modifications were done. For example, ‘State Parks have a peaceful/serene image’ was changed to ‘The state park has a peaceful/serene image’. Additionally, demographic profile such as educational qualification and income was added to the survey. For Korea, the English version of the survey was translated into the Korean language based on expert advice. The final survey was circulated using social media channels and email. Three social media channels—LinkedIn, Facebook, and Instagram—were used to share the survey on popular networks such as outdoor, recreation, and state park-related organizations and associations. Additionally, an online survey link was emailed to different groups of people in the authors’ network in each country. The respondents were filtered using two selection criteria: (1) the person should have visited a state park or similar outdoor location at least once in the past three years and (2) She/he should be above 18 years of age. It took more than 10 months during 2020 and 2021 to collect data from the three countries. After several reminders and repeated links submitted on the three social media platforms, a sufficient number of usable responses (812) were received that were adequate for data analysis.

### Sample characteristics

This section of the paper describes the demographics of respondents (those individuals who completed the survey) including gender, age, education, and household income. As shown in Table 2, the majority of survey respondents were female, regardless of the country of origin. On average, the median age of most respondents (76.7%) fell between 20 and 49 years. Though the majority of respondents (46.7%) reflected Caucasian or White, that is because Ethnicity was removed from the questionnaire in India due to no applicability. Those respondents were categorized as ‘Others’ or ‘Asian’. An almost equal number of respondents were graduate, undergraduate, and high school degree holders. Finally, all 812 respondents represented all income categories. Thus, as discussed above, the respondents were well spread across all demographic categories, making it a good representative sample for analysis.

**Table 2:** Respondents’ demographic information (*n* = 812).

Characteristics		Frequency	%
Gender	Male	336	41.4
	Female	476	58.6
Age	Under 20	71	8.7
	20–29	339	41.7
	30–39	141	17.4
	40–49	143	17.6
	50–59	78	9.7
	Over 60	40	4.9
Ethnic background	Black	10	1.2
	Asian	212	26.2
	Hispanic	10	1.2
	Caucasian/White	379	46.7
	Other	201	24.7
Education	Less than high school degree	2	0.2
	High school degree	67	8.3
	Some college	261	32.1
	College graduate	236	29.1
	Graduate degree	246	30.3
Annual household income	Under $24,999	112	13.8
	$25,000–$39,999	133	16.4
	$40,000–$54,999	127	15.6
	$55,000–$69,999	106	13.1
	$70,000–$84,999	129	15.8
	$85,000–$99,999	90	11.1
	Over $100,000	115	14.2

## Result and findings

### Data quality testing

A confirmatory factor analysis (CFA) was performed using the maximum likelihood estimation method to prove the reliability and validity of the presented scale. The analysis results are as follows. First, the model fit of the measurement model was acceptable (*χ*^2^ = 1013.050, df = 246, *p* < 0.001, *χ*^2^/df = 4.118, CFI = 0.925, TLI = 0.908, NFI = 0.904, and RMSEA = 0.062). Hence, it can be said that the model fit of the measurement model is at a statistically acceptable level. Next, the average variance extracted (AVE) and composite reliability (CR) were checked to verify the convergent validity and internal consistency. When the AVE value is 0.5 or more and the CR value is 0.7 or more, it can be said that there is no problem with the convergent validity and internal consistency of the measurement variables (Hair et al., 2010). Results of the analysis showed that the CR values ranged from 0.782 to 0.939, while the AVE values ranged from 0.547 to 0.838. Therefore, we can say that a statistically significant level of convergent validity and internal consistency has been secured. Finally, tests for discriminant validity were conducted to verify the differentiation between constructs. If the AVE value is greater than the squared value of the correlation coefficient, there is no problem with the discriminant validity (Hair et al., 2010). As a result of the analysis, it was found that the AVE value was larger than the squared value of the correlation coefficient between the variables presented in this study. Accordingly, the discriminant validity between constructs was evident. Table 3 includes the details of the findings.

**Table 3:** Confirmatory factor analysis results and between-construct correlations.

	1	2	3	4	5	6	7
1. FI	1.000						
2. SI	0.681^a^ (0.463)^b^	1.000					
3. EI	0.666 (0.443)	0.695 (0.483)	1.000				
4. P	0.702 (0.492)	0.705 (0.497)	0.664 (0.440)	1.000			
5. A	0.715 (0.511)	0.632 (0.399)	0.702 (0.492)	0.675 (0.492)	1.000		
6. PI	0.558 (0.311)	0.552 (0.304)	0.672 (0.451)	0.572 (0.327)	0.602 (0.362)	1.000	
7. RI	0.632 (0.399)	0.689 (0.474)	0.697 (0.485)	0.690 (0.476)	0.637 (0.405)	0.526 (0.276)	1.000
CR (AVE)	0.782 (0.547)	0.812 (0.599)	0.859 (0.605)	0.939 (0.838)	0.865 (0.682)	0.847 (0.735)	0.855 (0.624)
Mean (SD)	4.101 (0.635)	3.394 (0.677)	3.815 (0.668)	4.140 (0.682)	3.777 (0.757)	3.814 (0.855)	4.127 (0.682)

Goodness-of-fit statistics: *χ*^2^ = 1013.050, df = 246, *p* < 0.001, *χ*^2^/df = 4.118, RMSEA = 0.062, CFI = 0.925, NFI = 0.904, TLI = 0.908.

*FI* functional image, *SI* symbolic image, *EI* experiential image, *P* pleasure, *A* arousal, *PI* place identity, *RI* revisit intention.

^a^Correlations between variables are below the diagonal.

^b^Squared correlations between variables are within parentheses.

### Hypotheses testing

**Fig. 2:**
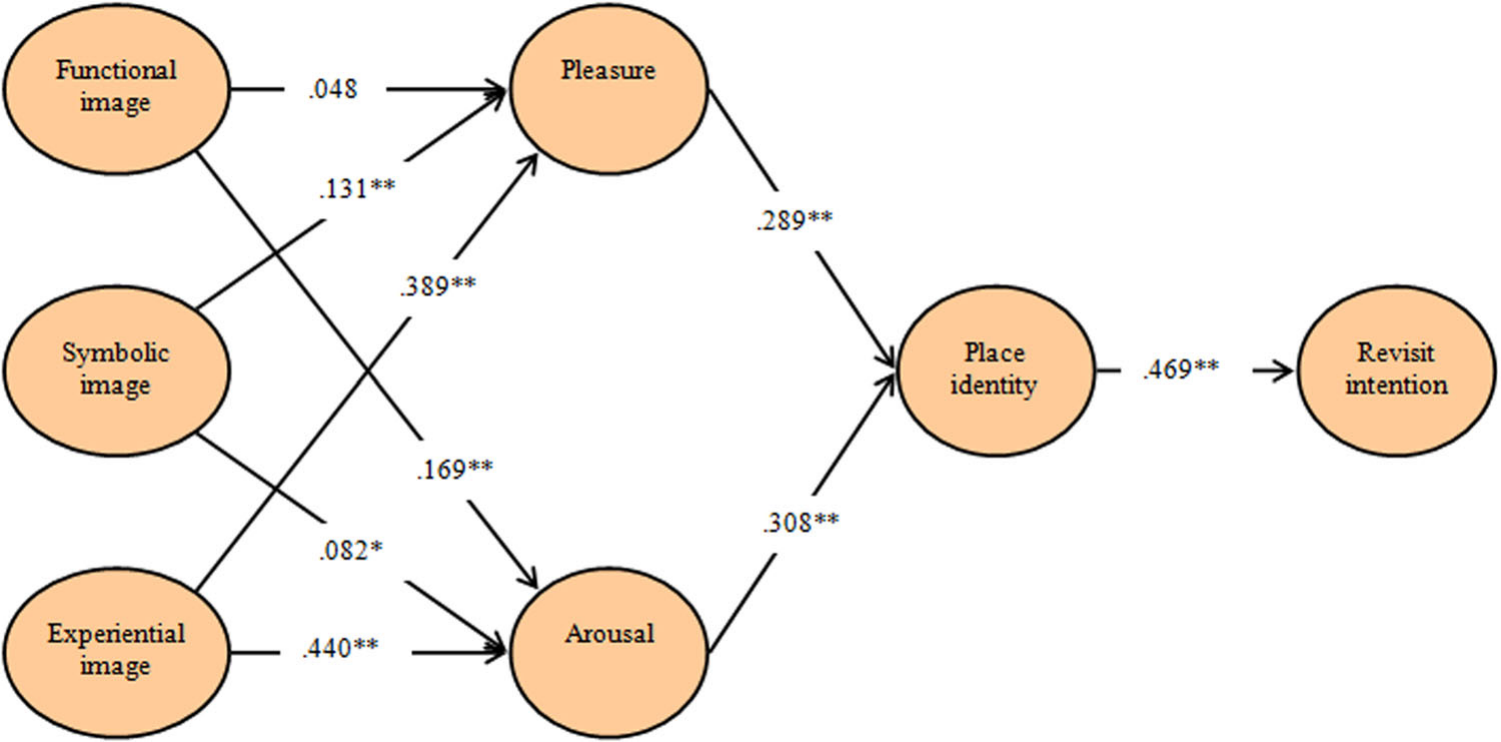
Results of the hypotheses tests.

In the present research, a total of 10 hypotheses were evaluated to confirm the relationship between the presented variables. A regression analysis was performed to test these hypotheses using SPSS 22. To test Hypotheses 1, 2, and 3, the relationships between the functional, symbolic, and experiential images of pleasure were examined. As a result, it was found that the functional image (H1: *β* = 0.048, *p* > 0.05) did not have a statistically significant effect, whereas the symbolic image (H2: *β* = 0.131, *p* < 0.01) and experiential image (H3: *β* = 0.389, *p* < 0.01) were found to have a statistically significant effect on pleasure. Next, to verify Hypotheses 4–6, the relationships between the functional, symbolic, and experiential images on arousal were examined. Results of the analysis showed that the functional image (H4: *β* = 0.169, *p* < 0.01), symbolic image (H5: *β* = 0.082, *p* < 0.01), and experiential image (H6: *β* = 0.440, *p* < 0.01) had a statistically significant effect on arousal. Then, to test Hypotheses 7 and 8, the relationships between pleasure and arousal on place identity were examined. Results of the analysis showed that both pleasure (H7: *β* = 0.289, *p* < 0.01) and arousal (H8: *β* = 0.308, *p* < 0.01) had a statistically significant effect on place identity. Finally, to test Hypothesis 9, the relationship between place identity and revisit intention was examined, which found that place identity (H9: *β* = 0.469, *p* < 0.01) had a statistically significant effect on revisit intention. In summary, if we look at the results of the hypotheses tests, it can be seen that while Hypothesis 1 was not accepted, Hypotheses 2–9 were all accepted. Detailed results are shown in Fig. 2 and Table 4.

**Table 4:** Results of the regression analysis.

Equations (*R*²) (Adjusted *R*²)	Independent variable → dependent variable	Coefficient (standardized)	*t*-value
Equation 1 (*R*² = 0.263) (Adjusted *R*² = 0.261)	FI → P	0.048	1.173
	SI → P	0.131	3.261**
	EI → P	0.389	9.285**
Equation 2 (*R*² = 0.383) (Adjusted *R*² = 0.381)	FI → A	0.169	4.474**
	SI → A	0.082	2.247*
	EI → A	0.440	11.457**
Equation 3 (*R*² = 0.295) (Adjusted *R*² = 0.293)	P → PI	0.289	7.423**
	A → PI	0.308	7.919**
Equation 4 (*R*² = 0.220) (Adjusted *R*² = 0.219)	PI → RI	0.469	15.112**

*FI* functional image, *SI* symbolic image, *EI* experiential image, *P* pleasure, *A* arousal, *PI* place identity, *RI* revisit intention.

*p < 0.05, **p < 0.01.

To demonstrate the moderating influence of national culture, a moderated regression analysis was conducted based on Baron and Kenny’s (1986) suggested procedures. The results of the analysis of Hypothesis 10a-i showed a moderating effect of national culture on the all the relationships. First, the moderating effect of national culture was examined in relation to the effects of functional, symbolic, and experiential image on pleasure. Results of the analysis showed a moderating effect of functional image (the United States [US]: predictor*moderator = −0.167, ∆*R*² = 0.000, *p* > 0.05; India: predictor*moderator = −0.546, ∆*R*² = 0.006, *p* < 0.01; Korea: predictor*moderator = 0.579, ∆*R*² = 0.010, *p* < 0.01), symbolic image (the US: predictor*moderator = 0.008, ∆*R*² = 0.000, *p* > 0.05; India: predictor*moderator = −0.147, ∆*R*² = 0.001, *p* > 0.05; Korea: predictor*moderator = 0.259, ∆*R*² = 0.002, *p* < 0.01), and experiential image (the US: predictor*moderator = −0.031, ∆*R*² = 0.000, *p* > 0.05; India: predictor*moderator = −0.182, ∆*R*² = 0.006, *p* > 0.05; Korea: predictor*moderator = 0.111, ∆*R*² = 0.000, *p* > 0.05). Next, the moderating effect of national culture was examined in relation to the effects of a functional, symbolic, and experiential images on arousal. Results of the analysis showed a moderating effect of a functional image (the US: predictor*moderator = 0.315, ∆*R*² = 0.002, *p* > 0.05; India: predictor*moderator = −0.457, ∆*R*² = 0.004, *p* < 0.01; Korea: predictor*moderator = 0.318, ∆*R*² = 0.003, *p* < 0.01), symbolic image (the US: predictor*moderator = 0.551, ∆*R*² = 0.007, *p* < 0.01; India: predictor*moderator = −0.205, ∆*R*² = 0.001, *p* > 0.05; Korea: predictor*moderator = 0.005, ∆*R*² = 0.000, *p* > 0.05), and experiential image (the US: predictor*moderator = 0.193, ∆*R*² = 0.001, *p* > 0.05; India: predictor*moderator = –0.505, ∆*R*² = 0.006, *p* < 0.01; Korea: predictor*moderator = 0.191, ∆*R*² = 0.001, *p* > 0.05). Next, the moderating effect of national culture was analyzed on the relationships between pleasure and arousal on place identity. Results of the analysis showed a moderating effect for pleasure (the US: predictor*moderator = 0.021, ∆*R*² = 0.000, *p* > 0.05, India: predictor*moderator = 0.085, ∆*R*² = 0.001, *p* > 0.05, Korea: predictor*moderator = −0.078, ∆*R*² = 0.000, *p* > 0.05) and arousal (the US: predictor*moderator = −0.099, ∆*R*² = 0.001, *p* > 0.05; India: predictor*moderator = −0.010, ∆*R*² = 0.000, *p* > 0.05; Korea: predictor*moderator = −0.036, ∆*R*² = 0.000, *p* > 0.05). Finally, the moderating effect of national culture on the relationship between place identity and revisit intention was examined. The results showed a moderating effect in the US (predictor*moderator = −0.234, ∆*R*² = 0.002, *p* > 0.05), India (predictor*moderator = 0.045, ∆*R*² = 0.000, *p* > 0.05), and Korea (predictor*moderator = 0.331, ∆*R*² = 0.006, *p* < 0.01). Therefore, it can be said that Hypotheses 10a-i presented in this study were partially accepted. Detailed results are shown in Table 5a–d.

**Table 5:** Hierarchical regression analysis for moderating role of national culture: (a) pleasure, (b) arousal, (c) place identity, and (d) revisit intention.

	P^a^				
	Step 1	Step 2	Step 3-1	Step 3-2	Step 3-3
(a)					
Independent variable					
FI	0.387** (USA)	0.391** (USA)	0.406** (USA)	0.391** (USA)	0.391** (USA)
	0.387** (India)	0.413** (India)	0.465** (India)	0.410** (India)	0.408** (India)
	0.387** (Korea)	0.380** (Korea)	0.299** (Korea)	0.372** (Korea)	0.376** (Korea)
SI	0.118** (USA)	0.120** (USA)	0.117** (USA)	0.119** (USA)	0.119** (USA)
	0.118** (India)	0.150** (India)	0.145** (India)	0.164** (India)	0.151** (India)
	0.118** (Korea)	0.131** (Korea)	0.120** (Korea)	0.096 (Korea)	0.128** (Korea)
EI	0.338** (USA)	0.338** (USA)	0.342** (USA)	0.338** (USA)	0.343** (USA)
	0.338** (India)	0.307** (India)	0.294** (India)	0.308** (India)	0.323** (India)
	0.338** (Korea)	0.314** (Korea)	0.315** (Korea)	0.315** (Korea)	0.303** (Korea)
National culture		−0.016 (USA)	0.143 (USA)	−0.024 (USA)	0.013 (USA)
		0.115 (India)	0.663 (India)	−0.263 (India)	0.294 (India)
		−0.089 (Korea)	−0.666 (Korea)	−0.343 (Korea)	−0.200 (Korea)
FI * National culture			−0.167 (USA)	–	–
			−0.546** (India)		
			0.579** (Korea)		
SI * National culture				0.008 (USA)	–
				−0.147 (India)	
				0.259* (Korea)	
EI * National culture					−0.031 (USA)
					−0.182 (India)
					0.111 (Korea)
*R*²	0.544 (USA)	0.545 (USA)	0.545 (USA)	0.545 (USA)	0.545 (USA)
	0.544 (India)	0.556 (India)	0.563 (India)	0.557 (India)	0.557 (India)
	0.544 (Korea)	0.552 (Korea)	0.562 (Korea)	0.554 (Korea)	0.552 (Korea)
∆*R*²	–	0.000 (USA)	0.000 (USA)	0.000 (USA)	0.000 (USA)
		0.012 (India)	0.006 (India)	0.001 (India)	0.001 (India)
		0.007 (Korea)	0.010 (Korea)	0.002 (Korea)	0.000 (Korea)

	A^a^				
	Step 1	Step 2	Step 3-1	Step 3-2	Step 3-3
(b)					
Independent variable					
FI	0.169** (USA)	0.189** (USA)	0.161** (USA)	0.202** (USA)	0.192** (USA)
	0.169** (India)	0.209** (India)	0.253** (India)	0.204** (India)	0.195** (India)
	0.169** (Korea)	0.163** (Korea)	0.119** (Korea)	0.163** (Korea)	0.156** (Korea)
SI	0.082* (USA)	0.091* (USA)	0.096** (USA)	0.024 (USA)	0.095** (USA)
	0.082* (India)	0.131** (India)	0.127** (India)	0.151** (India)	0.134** (India)
	0.082* (Korea)	0.094** (Korea)	0.088** (Korea)	0.093* (Korea)	0.089* (Korea)
EI	0.440** (USA)	0.440** (USA)	0.433** (USA)	0.433** (USA)	0.406** (USA)
	0.440** (India)	0.393** (India)	0.382** (India)	0.394** (India)	0.438** (India)
	0.440** (Korea)	0.419** (Korea)	0.419** (Korea)	0.419** (Korea)	0.400** (Korea)
National culture		−0.078 (USA)	−0.378 (USA)	−0.604 (USA)	−0.261 (USA)
		0.177 (India)	0.635 (India)	0.383 (India)	0.674 (India)
		−0.080 (Korea)	−0.397 (Korea)	−0.084 (Korea)	−0.271 (Korea)
FI * National culture			0.315 (USA)	–	–
			−0.457* (India)		
			0.318* (Korea)		
SI * National culture				0.551** (USA)	–
				−0.205 (India)	
				0.005 (Korea)	
EI * National culture					0.193 (USA)
					−0.505** (India)
					0.191 (Korea)
*R*²	0.383 (USA)	0.388 (USA)	0.390 (USA)	0.395 (USA)	0.389 (USA)
	0.383 (India)	0.411 (India)	0.415 (India)	0.412 (India)	0.417 (India)
	0.383 (Korea)	0.389 (Korea)	0.392 (Korea)	0.389 (Korea)	0.390 (Korea)
∆*R*²	–	0.005 (USA)	0.002 (USA)	0.007 (USA)	0.001 (USA)
		0.028 (India)	0.004 (India)	0.001 (India)	0.006 (India)
		0.006 (Korea)	0.003 (Korea)	0.000 (Korea)	0.001 (Korea)

	PI^a^			
	Step 1	Step 2	Step 3-1	Step 3-2
(c)				
Independent variable				
P	0.289** (USA)	0.312** (USA)	0.309** (USA)	0.310** (USA)
	0.289** (India)	0.313** (India)	0.307** (India)	0.313** (India)
	0.289** (Korea)	0.277** (Korea)	0.291** (Korea)	0.278** (Korea)
A	0.308** (USA)	0.303** (USA)	0.303** (USA)	0.324** (USA)
	0.308** (India)	0.208** (India)	0.280** (India)	0.280** (India)
	0.308** (Korea)	0.301** (Korea)	0.300** (Korea)	0.306** (Korea)
National culture		−0.087 (USA)	−0.108 (USA)	0.006** (USA)
		0.170 (India)	0.085 (India)	0.179** (India)
		−0.071 (Korea)	0.008 (Korea)	−0.035** (Korea)
P * National culture			0.021 (USA)	–
			0.085 (India)	
			−0.078 (Korea)	
A * National culture				−0.099 (USA)
				−0.010 (India)
				−0.036 (Korea)
*R*²	0.295 (USA)	0.302 (USA)	0.302 (USA)	0.303 (USA)
	0.295 (India)	0.323 (India)	0.324 (India)	0.323 (India)
	0.295 (Korea)	0.300 (Korea)	0.300 (Korea)	0.300 (Korea)
∆*R*²	–	0.007 (USA)	0.000 (USA)	0.001 (USA)
		0.028 (India)	0.001 (India)	0.000 (India)
		0.005 (Korea)	0.000 (Korea)	0.000 (Korea)

	RI^b^		
	Step 1	Step 2	Step 3
(d)			
Independent variable			
PI	0.469** (USA)	0.465** (USA)	0.515** (USA)
	0.469** (India)	0.504** (India)	0.501** (India)
	0.469** (Korea)	0.452** (Korea)	0.396** (Korea)
National culture		0.229 (USA)	0.451** (USA)
		−0.190 (India)	−0.233 (India)
		−0.087 (Korea)	−0.414 (Korea)
PI * National culture			−0.234 (USA)
			0.045 (India)
			0.331** (Korea)
*R*^2^	0.220 (USA)	0.272 (USA)	0.275 (USA)
	0.220 (India)	0.255 (India)	0.255 (India)
	0.220 (Korea)	0.227 (Korea)	0.234 (Korea)
∆*R*^2^	–	0.052 (USA)	0.002 (USA)
		0.035 (India)	0.000 (India)
		0.007 (Korea)	0.006 (Korea)

^a^*FI* functional image, *SI* symbolic image, *EI* experiential image, *P* pleasure, *A* arousal, *PI* place identity, *RI* revisit intention.

^b^*NEQ* nature environmental quality as NBS, *TSMH* traveler self-rated mental health, *TEWB* traveler emotional well-being, *LS* life satisfaction, *BI* behavior intention.

*p < 0.05, **p < 0.01.

## Discussion

Before summarizing the findings of this study and discussing its implications, it is important to know the limitations. First, the results should not be interpreted to be representative of all tourists from India, Korea, and US, due to the highly delimited nature of the sample. Second, we have interpreted the results in the context of the national culture of the three countries, but while there is no evidence as to which interpretation is accurate, it is our belief based on past research, knowledge (being part of the culture), and expertise that the interpretations are close to true. Third, the methodology used in this study may be limited in assessing cultural aspects, which were based on Hofstede’s cultural dimensions. No direct questions on culture were asked in the survey. Future studies could include cultural questions and utilize other models such as Schwartz’s cultural value orientation and GLOBE’s approach (Schwartz, 1994).

Even though this topic is crucial, the impact of brand image on state park visitors’ decision-making has been hardly examined. Almost none of the studies have investigated the associations among the multiple dimensions of state park image (functional, symbolic, and experiential), pleasure, arousal, place identity, and revisit intention, and especially there has been no investigation of the influence of national culture on these relationships. This study is the first of its kind to fill the above-mentioned gaps in the literature.

As demonstrated in the results section, the theoretical constructs utilized in this study play an important role in state park visitors’ decision-making process. Image, especially symbolic and experiential images, was identified to be of importance as all its subsequent constructs (i.e., pleasure, arousal, place identity, and visitors’ revisit intention) are significant and have a positive direct function of them. Place identity was found to be influential in generating visitors’ revisit intention to the state parks. Further, the symbolic and experiential image (barring functional image)—emotions (pleasure and arousal)—place identity—revisit intention relationships were found to have an influence on revisit intention. Finally, it was found that national culture does influence the relationships between the different variables studied in this study. Thus, the research objectives developed in this study on the state park sector were wholly attained.

Symbolic images and experiential images were found to be important in directly eliciting pleasure and arousal. In addition, these factors, which are key constituents of state park image, were identified to be significant contributors to boosting visitors’ attachment to the place and their revisit intention. As per branding and advertising research, non-experiential images such as functional are influenced by advertising more effectively as compared to sensory evaluations (Orth et al., 2012; Drolet and Aaker, 2002; Wright and Lynch, 1995), while symbolic images are influenced by product experience (Deighton, 1984; Kempf and Smith, 1998). Thus, the reason for the functional image not influencing arousal may be attributed to low or no advertising of state parks’ functional attributes. Given this, to enhance visitor emotions and attachment to the state parks and to ensure their revisit, it is essential for state park administrators/managers and policymakers to boost their state park’s image (functional, symbolic, and experiential). To position a symbolic image, state park managers should convey a symbolic concept by communicating the symbolic aspect of the state park. Marketing and promotional material of state parks should highlight the symbolic benefits of visiting the state parks such as peaceful and serene, environmentally friendly, sustainable, nature, landscapes, and even cultural heritage, depending on the state park’s location (Jepson and Sharpley, 2015; Kneafsey, 2000; López-Sanz et al., 2021b; Szubert et al., 2021)

To position experiential image, positioning strategies should communicate the state park’s effect on effective stimulation. Like other places, state parks may provide significant meanings, intentions, and purposes for visitors. One way to establish an experiential image is to deliver an enjoyable, pleasant, relaxing, rejuvenating, and revitalizing environment and provide a sense of wellness, meditation, and knowledge enrichment. Creating ‘local knowledge’ can especially help in attracting tourists via place identity (Kneafsey, 2000) and image (López-Sanz et al., 2021a, 2021c). State park managers are recommended to apportion a part of the budget on tangible cues (e.g., visitors’ center, welcome/reception office, rest areas, and interactive website), which have been proven to enhance visitor emotions and feelings positively (Han et al., 2019).

Findings from this study validate the past research results. For example, results verified that brand image leads to revisiting intentions (Han et al., 2019). Specifically, symbolic (*β* = 0.131, *p* < 0.01; *β* = 0.082, *p* < 0.01) and experiential (*β* = 0.389, *p* < 0.01; *β* = 0.440, *p* < 0.01) images have relative importance in inducing emotions: pleasure and arousal, respectively. These results are in line with previous research studies, which found the role of brand image in influencing emotions (Hosany et al., 2017). Similarly, emotions, pleasure (*β* = 0.289, *p* < 0.01) and arousal (*β* = 0.308, *p* < 0.01), positively affect place identity/attachment, and place identity/attachment (*β* = 0.469, *p* < 0.01) leads to revisit intentions among state park visitors. A vast amount of literature indicates that visitors’ emotions are essential for attachment to a destination (e.g., Yan and Halpenny, 2019) and place identity/attachment leads to behavioral intention to revisit a destination (e.g., Patwardhan et al., 2020). The study interestingly reveals that functional image does not affect the pleasure in state park context, which may be attributed to various reasons. For example, state parks’ functional or external consumption attributes (e.g., accommodations, facilities, etc.) are not the motivations for visitors nor are they considered as given, probably due to destinations' nature (natural and wilderness). This confirms that state parks are unique destinations in many ways, and therefore more research is needed to understand their visitors’ needs and motivations and to market them.

All hypotheses but one are statistically confirmed, but the strength of different relationships varies, as seen in the structural model, based on different construct combinations. There is enough evidence in the past literature but on different constructs. The relationship between PI → RI is the strongest (path 0.469; t 15.112). This result is in line with recent studies on place attachment (e.g., Su et al., 2011) confirming that place identity/attachment determines to revisit intention to state parks. It is worth exploring whether they visit the same state park again or a different one. The relationship between EI → A is the second strongest (path 0.440; t 11.457) followed by EI → P (path 0.389; t 9.285), which concurs with a plethora of research studies on brand image and emotions: pleasure and arousal (e.g., Hosany et al., 2017) establishing that experiential image has relatively most influence on consumer/visitors’ emotions (discussed earlier in this paper). Next in line is P→PI (path 0.289; t 7.423), validating the results from previous studies (e.g., Morgan 2010) and confirming that pleasurable emotions generated during visits to state parks help develop place identity/attachment. It is worth noting that arousal has a relatively higher influence on emotions to attach visitors to the state park, which may be attributed to the personality or type of person s/he is (Kuppens, 2008) or the type of place (Scannell and Gifford, 2014). Therefore, further research to explore this aspect is suggested.

Arousal is the most significant independent construct (R^2^ of 0.383) followed by place identity/attachment (*R*^2^ of 0.295) and pleasure (*R*^2^ of 0.263). Though the combined effect of six constructs has a lower determination power of 22%, it may be attributed to post-visit data collection that reduces intention to revisit (Santos et al., 2017) or due to the pandemic (COVID-19) restrictions because of which respondents did not intend to revisit at all.

An important objective of this study was to assess the moderating effect of national culture on the relationships in the structural model (H10a-i). The results are helpful to explain how national culture affects these relationships or not. This paper finds that national culture moderates the relationships between brand image and emotions and between place identity/attachment and revisit intention.

National culture was found to moderate the relationship of functional image with pleasure. It is interesting to note that in the case of India, it negatively moderates this relationship (Coef. = −0.546, *p* < 0.1), but positively so in the case of Korea (Coef. = 0.579, *p* < 0.1), while no significant relationship was found in the case of the US. This is because Koreans may visualize a state park as being a natural, inexpensive (long-term orientation), safe place (higher uncertainty avoidance) that may enhance life quality (feminine) and bring happiness when planning to visit with their group members (collective society). On the contrary, national culture in the case of India shows a negative relationship because India is a moderate collectivist society, which may attribute to less inclination towards pro-environmental behavior. Being a low uncertainty avoidance culture, Indian visitors may perceive state parks as not something new and unique. A moderately long-term oriented and masculine society may mean less preference for satisfaction than usefulness, success, or materialistic gain. This is in line with past research on the influence of culture on consumer behavior (e.g., Bearden et al., 2006; Chen, 2000; Crotts and Erdmann, 2000; Kim and Choi, 2005; McCarty and Shrum, 2001; Money and Crotts, 2003; Reisinger and Crotts, 2009).

For symbolic image, national culture is found to positively moderate its relationship with pleasure in the case of Korea (Coef. = 0.259, *p* < 0.5), while having no significant effect on India and US. As a state park, symbolic images portray a peaceful, sustainable, and sometimes cultural heritage image, the national culture in Korea shows a positive influence on pleasure. This is because Koreans value peace, nature, loves, and cares for group members including the environment as characterized by very low individualism or very high collectivism (Farahani and Mohamed, 2011; Kim and Choi, 2005; McCarty and Shrum, 2001).

National culture is found to moderate the relationship of functional image with arousal. In the case of India, it negatively moderates this relationship (Coef. = −0.457, *p* < 0.5), while it positively does so in the case of Korea (Coef. = 0.318, *p* < 0.5). This is consistent with the results with pleasure (emotions) confirming that the interaction effect in the case of these cultures (countries) is consistent for both types of emotions: pleasure and arousal. No significant effect was found in the case of the US.

National culture is found to positively moderate the relationship of symbolic image with arousal in the case of the US (Coef. = 0.551, *p* < 0.1), while no significant effect was found on India and Korea. While the symbolic image state parks portrays a peaceful, sustainable, and sometimes cultural heritage image, it can be interpreted by visitors as new and unique from low uncertainty avoidance cultures (Reisinger and Crotts, 2009) such as the US, and challenging and adventurous because of their individualistic nature (De Mooij, 2004; Hofstede, 2001). Finally, it can be logically assumed that being high in indulgence and masculinity cultural traits representing free gratification and achievement, US visitors expressed higher affinity with excitement, astonishment, and delighted feelings representing arousal (emotions) towards state park symbolic images (Koc et al. 2017).

National culture is found to negatively moderate the relationship of experiential image with arousal in case of India (Coef. = −0.505, *p* < 0.1), while there was no significant effect on Korea and US. This can be attributed to the low uncertainty avoidance culture of Indian visitors, who may perceive state parks as not something new and unique. They are less inclined towards environment or nature (Chen, 2000) because of moderate individualistic culture. Plus, being from a moderately long-term oriented and masculine society, Indians aspire for success and materialistic gains rather than revitalization and satisfaction (Reisinger and Crotts, 2009).

For place identity, national culture is found to positively moderate its relationship with revisit intention in the case of Korea (Coef. = 0.331, *p* < 0.1), while no significant effect was found in the case of India and the US. Korean culture is identified by very high long-term orientation and uncertainty avoidance traits, slightly high to moderate power distance and masculinity, and low on indulgence and very low on individualism. Koreans value peace, nature, love, and care for group members including the environment (Farahani and Mohamed, 2011; Kim and Choi, 2005; McCarty and Shrum, 2001) as explained above.

### Implications of the study

The findings of this study have both theoretical and managerial implications. This study demonstrates that national culture is an important determinant that influences state park visitors’ decision-making. Therefore, it is recommended that state park managers should develop marketing strategies based on the national culture of their target segments. Effective marketing strategies should be developed using appropriate brand images (functional, symbolic, and experiential) to attract target visitors. For example, visitors from cultures such as Korea can be attracted by creating images portraying opportunities to socialize, escape from routine, outdoor activities, green natural stress-free environment, accommodation facilities, and peaceful, sustainable, and cultural heritage image because of their national culture. On the contrary, for cultures such as the US, state parks should create an image portraying uniqueness, adventure, excitement, challenge, free gratification, and a sense of achievement. Lastly, for cultures like India, state parks should portray an image to show usefulness, success, or materialistic gain to attract visitors. Thus, this study provides strong evidence of cultural differences within broad cultural categories such as Western and Asian, which should be used for developing specific marketing and promotional strategies to target state park visitors. This study, therefore, is an important contribution to the existing literature on cross-cultural marketing within the broad tourism discipline.

### Limitations of the study

This study has certain limitations that offer future research avenues. First, the study was conducted in a state park context in USA, India, and Korea. The scenario and manipulations were used assuming the respondents visited atleast one state park in recent past. A future study can conduct similar research in different state parks in different countries such as Brazil, South Africa, and Australia. Moreover, a major caveat is associated with sampling design. Data was collected via social media channels and by emails, which can be categorized as convenience sampling. The survey was though circulated multiple times on popular networks related to outdoor, recreation, and state park related organizations and associations to strengthen the validity of the results. Therefore, future researchers are encouraged to replicate this study using the probability sampling method to enhance the generalizability of current findings and use social media channels such as Twitter and not Facebook. Finally, this study used place identity to assess attachment with a place, assuming that people do not have dependence or functional attachment with state parks, however, future studies may consider multidimensional approach to place attachment including place identity and place dependence.

## Supplementary information

Supplementary information

## Data Availability

The datasets generated during and/or analyzed during the current study are available from the corresponding author on reasonable request.
