# Supplementary material for: Exploring the impact of functional, symbolic, and experiential image on approach behaviors among state-park tourists from India, Korea, and the USA
Source: Humanit Soc Sci Commun. 2023 Jan 27;10(1):36. doi: 10.1057/s41599-023-01527-y (PMC9881514; doi:10.1057/s41599-023-01527-y)
Supplement: Supplementary file 1 — Supplementary information [file 41599_2023_1527_MOESM1_ESM.docx]

**Supplementary Information**

| **MEASUREMENT ITEMS** |
| --- |
| **Functional image (Arklina et al., 2020; Paek et al., 2012)** |
| Visiting the State park relieves my stress and alleviates tension |
| Visiting the State park allows to socialize |
| Visiting the State park provides escape from daily routine |
| Variety of outdoor activities are provided at the State park |
| Accommodations and facilities (tourist information centers, bathrooms, etc.) at the State park are excellent |
| **Symbolic Image (Arklina et al., 2020; Kim & Kim, 2011)** |
| The State park has a peaceful/serene image |
| The State park has a sustainability image |
| The State Park has a cultural heritage image |
| **Symbolic Image (Arklina et al., 2020; Park et al., 1986)** |
| Visiting the State park changes my daily life/routine |
| Visiting the State park vitalizes my life |
| Visiting the State park enriches my knowledge |
| The State park visit allows me to experience the unique atmosphere |
| **Pleasure (Han et al., 2018; Song et al., 2015)** |
| I felt a ‘joyful’ and ‘cheerful’ emotion at the State park |
| I felt a ‘happy’ emotion at the State park |
| I felt a ‘satisfaction’ emotion at the State park |
| **Arousal (Han et al., 2018; Tsaur et al., 2007)** |
| I felt ‘excited/thrilled’ at the State park |
| I felt ‘astonished’ at the State park |
| I felt ‘elated/delighted’ at the State park |
| **Place identity (Groshong et al., 2019; Ispas et al., 2021; Wynveen et al., 2020)** |
| I feel a special connection to the State park and its green atmosphere |
| I identify strongly with the State park and its green atmosphere |
| **Revisit Intention (Arklina et al., 2020; Han et al., 2018)** |
| I will visit/revisit the State park next time |
| I will continue to visit the State park, even if it involves time |
| I intend to visit the State park continuously |
